# Evidence for Positive Selection within the *PgiC1* Locus in the Grass *Festuca ovina*

**DOI:** 10.1371/journal.pone.0125831

**Published:** 2015-05-06

**Authors:** Yuan Li, Björn Canbäck, Tomas Johansson, Anders Tunlid, Honor C. Prentice

**Affiliations:** Department of Biology, Lund University, Lund, Sweden; National University of Rosario, ARGENTINA

## Abstract

The dimeric metabolic enzyme phosphoglucose isomerase (PGI, EC 5.3.1.9) plays an essential role in energy production. In the grass *Festuca ovina*, field surveys of enzyme variation suggest that genetic variation at cytosolic PGI (PGIC) may be adaptively important. In the present study, we investigated the molecular basis of the potential adaptive significance of PGIC in *F*. *ovina* by analyzing cDNA sequence variation within the *PgiC1* gene. Two, complementary, types of selection test both identified PGIC1 codon (amino acid) sites 200 and 173 as candidate targets of positive selection. Both candidate sites involve charge-changing amino acid polymorphisms. On the homology-modeled *F*. *ovina* PGIC1 3-D protein structure, the two candidate sites are located on the edge of either the inter-monomer boundary or the inter-domain cleft; examination of the homology-modeled PGIC1 structure suggests that the amino acid changes at the two candidate sites are likely to influence the inter-monomer interaction or the domain-domain packing. Biochemical studies in humans have shown that mutations at several amino acid sites that are located close to the candidate sites in *F*. *ovina*, at the inter-monomer boundary or the inter-domain cleft, can significantly change the stability and/or kinetic properties of the PGI enzyme. Molecular evolutionary studies in a wide range of other organisms suggest that PGI amino acid sites with similar locations to those of the candidate sites in *F*. *ovina* may be the targets of positive/balancing selection. Candidate sites 200 and 173 are the only sites that appear to discriminate between the two most common PGIC enzyme electromorphs in *F*. *ovina*: earlier studies suggest that these electromorphs are implicated in local adaptation to different grassland microhabitats. Our results suggest that PGIC1 sites 200 and 173 are under positive selection in *F*. *ovina*.

## Introduction

The identification of the key genes and then the key mutations that underlie fitness variation is one of the central tasks in evolutionary biology [1]. Candidate genes that may be involved in fitness differences in natural populations of non-model species can often be proposed on the basis of information from earlier studies on model organisms [1]. One such candidate is the gene that codes for the dimeric enzyme phosphoglucose isomerase (PGI) (EC 5.3.1.9) [1]. PGI catalyzes the reversible isomerization between glucose-6-phosphate and fructose-6-phosphate, in the glycolytic pathway [2]. Variation in PGI activity is expected to affect the activity of the glycolytic pathway, which plays a central role in the production of energy and is therefore likely to be implicated in organisms’ adaptive responses to their environment.

High levels of variation in PGI enzyme electrophoretic mobility have been frequently reported, and significant correlations between PGI enzyme electromorphs and environmental variables, such as temperature, have been found in a wide range of organisms (reviewed in [3], [4]). Biochemical analyses in a number of species have demonstrated functional differences between PGI electromorphs (e.g. [5], [6]) that are consistent with the PGI electromorph-environment correlations in these species, suggesting that PGI itself may be the target of natural selection (e.g. [7], [8]).

Molecular evolutionary studies of the gene coding for PGI in both plants (e.g. [9], [10]) and animals (e.g. [11], [12]) often reveal a non-neutral pattern of DNA polymorphism, which is usually interpreted in terms of positive and/or balancing selection on PGI. Most of these studies propose particular charge-changing amino acid sites as the potential targets of selection (e.g. [13]). The potentially selected amino acid sites are usually enzyme electromorph-distinctive (e.g. [14]). Two studies [11], [15] involving homology-modeled 3-D PGI dimeric protein structure have shown that the potentially selected sites are located in the interface between the two monomers.

The majority of in-depth studies of the adaptive significance of PGI in natural populations have been carried out on animals [4], [16]. However, a possible adaptive role for PGI has also been proposed for a number of plant species, including the grass *Festuca ovina* L., which is the focus of the present study. Prentice et al. [17], [18] investigated PGI enzyme electromorph variation within populations of *F*. *ovina* in the steppe-like “alvar” grasslands on the Baltic island of Öland (Sweden). These grasslands are notable for their complex mosaic of different abiotic (edaphic) conditions, which is repeated in sites throughout the 26 000 ha area of alvar habitat in the southern part of the island. Using this naturally replicated study system, Prentice et al. [17] showed that, despite the fact that the species is wind-pollinated and outcrossing, with high levels of gene flow, enzyme electromorph frequencies at cytosolic PGI (PGIC) in samples of *F*. *ovina* were significantly related to local microhabitat variation—suggesting local adaptation. The fact that electromorph frequencies at PGIC changed, as predicted, after a nine-year experimental manipulation of the alvar habitat conditions [18], provided additional support for an adaptive role for PGIC variation in *F*. *ovina* [18].

In diploid [19] Swedish *F*. *ovina*, PGIC is coded for by two loci [20]: the *PgiC1* locus is present in all *F*. *ovina* individuals whereas the functional version of the *PgiC2* locus, which has been acquired from the genus *Poa* [21], [22], occurs in low frequencies in some populations [23]. The two most common PGIC enzyme electromorphs (EMs 1 & 2), are predominantly coded for by *PgiC1* (unpublished data), and show significant associations with fine-scale environmental variables in the Öland grasslands [17], [18].

The present study further explores the possible adaptive significance of the PGIC variation in *F*. *ovina* by examining the cDNA sequences of the *PgiC1* gene. We used two, complementary, types of method for the detection of positive selection within the *PgiC1* cDNA, and modeled the 3-D protein structure of PGIC1. Variation in the electrophoretic mobility of enzymes is predominantly a reflection of changes in molecular charge [3], [24]. Therefore, if enzyme variation in PGIC is adaptive in *F*. *ovina*, we predict: **(1)** that particular amino acid sites that involve charge-changing polymorphisms will be identified as being under positive selection; **(2)** that the location/s of these selected amino acid sites in the homology-modeled PGIC1 3-D protein structure, and the predicted modification of the local structure of the PGIC1 protein as a result of charge-changes at the selected sites, will indicate that the sites are likely to be functionally important; and **(3)** that the selected charge-changing polymorphisms will differentiate between the PGIC enzyme electromorphs that have earlier been shown to exhibit significant frequency differences between different microhabitats.

## Results

*PgiC1* cDNA, from 15 *F*. *ovina* individuals sampled from different microhabitats on the Baltic island of Öland (Sweden) (Table 1), was PCR-amplified, cloned and sequenced (in both forward and reverse directions). In total, we identified 30 *PgiC1* cDNA sequences (GenBank accession numbers: KF487737-KF487766) from the 15 analyzed individuals: these sequences belong to 22 haplotypes (Hap1-22; S1 File, S1 Table). In the analyzed material, a particular *PgiC1* haplotype may occur in several individuals, but the two *PgiC1* sequences from the same (diploid, [19]) individual always belong to two different haplotypes (S1 Table).

**Table 1:** *Festuca ovina* individuals analyzed.

Individual	Sample code	Sampling site	Microhabitat category	PGIC electromorph phenotype
1	AB7:1:2aIII	AB	7	1,2
2	TR6:2:5aIII	TR	6	2
3	PE2:1:2bIII	PE	2	1,4
4	BY2:2:2aIII	BY	2	1,2,4
5	BY7:2:2aII	BY	7	2,4
6	GL1:2:5aIII	GL	1	2,5
7	GL6:2:2aII	GL	6	2,4
8	PE1:1:4aII	PE	1	2,4,6
9	TR6:2:2a	TR	6	1,4
10	AB2:1:1aIII	AB	2	2
11	AB2:1:3aIII	AB	2	1,6
12	PE7:1:3aIII	PE	7	2
13	GL7:2:1aIII	GL	7	1,2
14	BY1:1:2aIII	BY	1	1,2
15	TR1:2:2aII	TR	1	2

**NOTES.—**Sample code is the collecting code for each individual. Sampling sites are located in the alvar grasslands of Öland, Sweden, from south to north: Albrunna (AB) 56° 19′ N/16° 25′ E, Penåsa (PE) 56° 26′ N/16° 27′ E, Gösslunda (GL) 56° 29′ N/16° 30′ E, Bårby alvar (BY) 56° 31′ N/16° 29′ E and Torrör (TR) 56° 34′ N/16° 34′ E. Microhabitat categories are 1 (low pH, moist), 2 (low pH, dry), 6 (high pH, dry) and 7 (high pH, moist). PGIC electromorph phenotypes were scored as in Prentice et al. [17], [18].

With the exception of Hap22, all identified *PgiC1* sequences cover 1 633 bp (nucleotide positions 19 to1 651) out of the 1 701 bp *F*. *ovina* full-length *PgiC1* cDNA sequence (as characterized by Vallenback, Ghatnekar and Bengtsson [22]), and translate into a polypeptide of 544 amino acid residues. An insertion of 113 bp between exon1 and exon2 was found in Hap22. This insertion is almost identical (1-bp difference) to intron 1 in the published *PgiC1* gene sequence with GenBank acc. no. HQ616103 [22]. Hence this insertion is likely to reflect incomplete splicing of the *PgiC1* precursor mRNA. Hap 22 was only present in individual 10 and, when the inserted intron sequence was removed, its sequence was identical to that of Hap10. Subsequent analyses were based on the 29 identified *PgiC1* sequences (excluding Hap22).

A high level of nucleotide variation was detected within the *PgiC1* gene. Alignment of the 29 *PgiC1* cDNA sequences revealed 89 mutations at 88 polymorphic sites. Twenty six of the polymorphic sites were singleton sites. Sixty seven of the mutations were synonymous and 22 were nonsynonymous. Three variants, all of which were synonymous, were found at nucleotide position 282. The total nucleotide diversity (*π*, [25]) was 0.01211, and Watterson’s estimator of the population mutation rate, *θ*_W_ [26] per site was 0.01372.

### Candidate targets of positive selection

Two complementary types of approach, one with a phylogenetic basis (HyPhy [27], [28] and PAML [29]) and one with a population genetics basis (omegaMap [30]), were used to test for positive selection at *PgiC1*. Together, these analyses suggest that codon (amino acid) sites 200 and 173 represent good candidate targets of positive selection.

A signal of positive selection was found for the non-recombinant cDNA sequence fragment spanning nucleotide positions 562–855, using the two nested tests (M1a + M2a and M7 + M8) in PAML. The “selection” models (M2a/M8) fit the data significantly better than the “neutral” models (M1a/M7) (Table 2), and the superior performance of the selection models in fitting the data was attributable to one codon site (200) that was a strong candidate for positive selection (Table 2). The candidateship of site 200 as a target of positive selection was also supported by omegaMap (Table 2) (the posterior probability of positive selection on site 200 is 1), and by the Random Effects Likelihood (REL) method in HyPhy (Table 2). In addition, the REL method also suggested positive selection on codon site 173 (Table 2), as did omegaMap and selection models M2a and M8 in PAML (Table 2). However, in omegaMap, the posterior probability for positive selection on site 173 is only 0.66, and in PAML the selection model and the neutral model gave similar results for the non-recombinant *PgiC1* cDNA sequence segment spanning nucleotide positions 196–561 (Table 2), where codon site173 is located.

**Table 2:** Tests for positive selection using *ω*-ratio tests: candidate amino acid sites identified by all the *ω*-ratio tests are underlined.

	Candidate targets (amino acid sites) of positive selection identified by			
*PgiC1* segment	PAML^b^ /M1a, M2a	PAML^b^ /M7, M8	HyPhy/REL^c^	omegaMap^d^
19–195^a^	(2*d*L = 0)	(2*d*L = 0)	53	
196–561^a^	109, 173 (2*d*L = 0)	109, 173 (2*d*L = 0.2)	**109**, **173**	
562–855^a^	**200** (2*d*L = 8*)	**200** (2*d*L = 9.2*)	**200**, **227**	
856–1650^a^	(2*d*L = 0)	(2*d*L = 0)		
19–1650				43, 46–53, 128, 145, 148–150, 170, 172, 173, **200**, 334, 423–425, 521

^a^All the PAML and HyPhy analyses were run on four non-recombinant *PgiC1* segments that are delimited on the basis of the three recombinant breakpoints (nucleotide positions 195, 561 and 855) identified by GARD.

^b^Within the PAML package, two nested tests, each including one neutral (M1a/M7) and one selection model (M2a/M8), were used to test for selection. Within each of the two nested tests, 2*d*L is the likelihood-ratio statistic used to test whether the selection model fits the data significantly better than the neutral model (df = 2).

Candidate targets of positive selection identified in M2a, M8 are those with posterior probabilities >50% in Bayes Empirical Bayes analyses. Sites with posterior probabilities >99% are indicated in bold text.

*: 0.01 < *p* < 0.05.

^c^The model REL within the Hyphy package was used to test for selection. Candidates targets of positive selection are those with Bayes factors > 50, candidates with Bayes factors larger than 500 are shown in bold text.

^d^Candidate targets of positive selection identified in omegaMap are those with posterior probabilities >50%. Sites with posterior probabilities >99% are shown in bold text.

The amino acid polymorphisms at both candidate sites 173 and 200 involve a charge change (Table 3). At site 173, two amino acid residues were detected in the 15 studied *F*. *ovina* individuals: one residue (Glu) has a negatively charged side chain, whereas the side chain of the other residue (Gln) is polar and uncharged. At site 200, one (Asp) of the three detected residues also has a negatively charged side chain, while the other two residues have either an aliphatic (Gly) or an uncharged polar side chain (Asn).

**Table 3:** Amino acid polymorphism among the 29 translated PGIC1 amino acid sequences from *F*. *ovina*.

				2	3					5		6				7	8	9	14	18		19	20	22
Hap	pI	Ind	Ind EM	43	47	48	49	53	64	109	128	145	149	150	170	173	200	227	334	423	425	452	474	521
Consensus				A	G	I	Y	A	K	H	Q	P	V	V	T	E	D	I	G	T	S	S	F	Q
Hap1	6.89	1	***1***,2	-	-	-	-	-	-	N	-	-	-	-	-	***E***	***D***	-	-	-	T	-	L	-
Hap1	6.89	9	***1***,4	-	-	-	-	-	-	N	-	-	-	-	-	***E***	***D***	-	-	-	T	-	L	-
Hap13	6.89	3	***1***,4	-	-	-	-	S	-	N	-	-	-	-	-	***E***	***D***	-	-	-	-	-	-	-
Hap14	6.89	3	***1***,4	-	-	-	-	S	-	N	-	-	-	-	-	***E***	***D***	L	-	-	-	-	-	-
Hap8	6.89	6	2,5	-	-	-	-	-	-	N	-	-	-	-	-	***E***	***D***	-	-	-	-	-	-	-
Hap3	6.93	9	***1***,4	-	-	-	-	-	R	-	-	-	-	-	-	***E***	***D***	-	-	-	-	-	-	-
Hap17	6.93	11	***1***,6	-	-	-	-	-	-	-	-	-	-	-	-	***E***	***D***	-	-	-	-	-	-	-
Hap20	6.93	13	***1***,2	-	-	-	-	-	-	-	-	-	-	-	-	***E***	***D***	-	-	-	-	-	-	-
Hap21	6.93	14	***1***,2	-	-	-	-	-	-	-	-	-	-	-	-	***E***	***D***	-	-	-	-	-	-	-
Hap5	6.96	4	***1***,2,4	-	-	V	H	S	-	-	-	-	-	-	-	***E***	***D***	-	-	A	-	-	-	-
Hap4	7.05	8	***2***,4,6	-	S	-	-	-	-	N	-	-	-	-	-	***E***	N	L	-	-	-	G	-	-
Hap16	7.05	11	1,6	-	-	-	-	-	-	N	-	-	-	-	-	***E***	N	-	-	-	-	-	-	-
Hap9	7.07	6	***2***,5	-	-	-	-	S	-	-	-	-	-	-	-	Q	***D***	-	-	A	-	-	-	-
Hap6	7.07	7	***2***,4	-	-	-	-	-	-	-	-	-	-	-	-	Q	***D***	-	-	A	-	-	-	-
Hap6	7.07	15	***2***	-	-	-	-	-	-	-	-	-	-	-	-	Q	***D***	-	-	A	-	-	-	-
Hap15	7.07	10	***2***	-	-	-	-	-	-	-	-	-	-	-	-	Q	***D***	-	A	-	-	-	-	-
Hap15	7.07	15	***2***	-	-	-	-	-	-	-	-	-	-	-	-	Q	***D***	-	A	-	-	-	-	-
Hap19	7.07	12	***2***	-	-	V	H	S	-	N	-	-	-	-	-	Q	***D***	-	-	-	-	-	-	-
Hap7	7.07	7	***2***,4	-	-	-	-	-	-	-	-	-	-	-	-	***E***	G	-	-	-	-	-	-	-
Hap7	7.07	13	1,***2***	-	-	-	-	-	-	-	-	-	-	-	-	***E***	G	-	-	-	-	-	-	-
Hap2	7.07	1	1,***2***	-	-	-	-	-	-	-	-	Q	-	-	-	***E***	G	-	-	-	-	-	-	-
Hap2	7.07	8	***2***,4,6	-	-	-	-	-	-	-	-	Q	-	-	-	***E***	G	-	-	-	-	-	-	-
Hap2	7.07	4	1,***2***,4	-	-	-	-	-	-	-	-	Q	-	-	-	***E***	G	-	-	-	-	-	-	-
Hap2	7.07	2	***2***	-	-	-	-	-	-	-	-	Q	-	-	-	***E***	G	-	-	-	-	-	-	-
Hap2	7.07	14	1,***2***	-	-	-	-	-	-	-	-	Q	-	-	-	***E***	G	-	-	-	-	-	-	-
Hap10	7.07	5	2,4	-	-	-	-	-	-	-	-	-	-	I	A	***E***	***D***	-	-	-	-	-	-	R
Hap18	7.07	12	***2***	-	-	-	-	-	-	-	-	-	-	-	-	***E***	N	-	-	-	-	-	-	-
Hap12	7.11	2	***2***	V	-	-	-	-	-	-	H	-	-	-	-	***E***	N	-	-	-	-	-	-	-
Hap11	7.21	5	2,4	-	-	-	-	-	-	N	-	-	I	-	-	Q	G	-	-	-	-	-	-	-

**NOTES.—**The exon number is shown in the first row of the table header. The vertically arranged numbers in the second row of the table header indicate the location of each polymorphic amino acid site. The first row beneath the table header is the consensus sequence, where each amino acid represents the most frequent residue in the alignment at each position. Each of the subsequent rows represents a single predicted amino acid sequence; for each sequence (apart from the consensus sequence), Hap = haplotype, pI = isoelectric point (predicted from the translated haplotype sequence using Protein calculator v3.3 http://www.scripps.edu/~cdputnam/protcalc.html), Ind = the *F*. *ovina* individual from which the sequence was derived, Ind EM = the PGIC enzyme electromorph phenotype (coded for by the *PgiC1* and *PgiC2* loci) of the individual. Apart from the consensus sequence, all the sequences are listed in ascending order of predicted pI values. Dashes (-) within a sequence indicate that the amino acid residue at a polymorphic site is the same as in the consensus sequence. The negatively charged residues at the selected polymorphic amino acid sites 173 and 200 are shown in bold italics. In the column Ind EM, bold italics indicate: EM 1 in the sequences with negatively charged residues at both sites 173 and 200; EM 2 in sequences with negatively charged residue at either sites 173 or 200, but not at both sites.

### The possible functional importance of the candidate targets for selection

**Fig 1:**
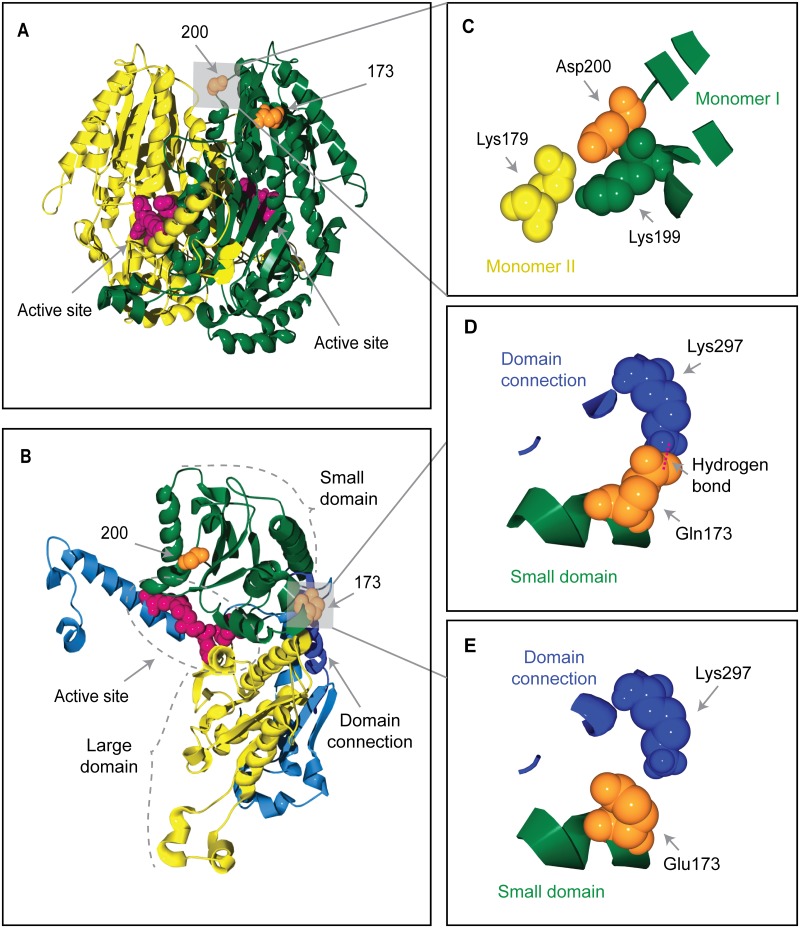
Homology-modeled 3-D structure of *F*. *ovina* PGIC1 and the candidate targets of positive selection. (A) A PGIC1 dimer, with the two monomers shown in green and yellow, respectively. The candidate sites 200 and 173 (space-filled, orange) are mapped onto one of the monomers. Site 200 is located on the edge of the inter-monomer boundary. Shown in the active sites are the four most conserved PGI residues (equivalent to Lys516, Glu360, His391 and Arg274 in *F*. *ovina*) [35] (space-filled, magenta). (B) A PGIC1 monomer showing that the candidate site 173 (space-filled, orange) is located on the edge of the cleft between the small (green) and large (yellow) domains. The active site is partially located within the cleft. The two domains are interconnected by a single polypeptide (Domain connection: dark blue). (C)-(E) Local structures showing that the charge changes at the candidate sites may affect the inter-monomer interaction or the domain-domain packing. (C) Shows all the residues occurring within a distance of 6Å from the candidate site 200 (space-filled, orange). The acidic Asp200 is adjacent to two basic residues: Lys199 (space-filled, green) is located in the same monomer (monomer I, green) as Asp200; Lys179 (space-filled, yellow) is in the opposite monomer (monomer II, yellow). (D) & (E) Show all the residues occurring within a distance of 6Å from the candidate site 173 (space-filled, orange) which is located on the small domain (green). Site 173 is close to Lys297 (space-filled, dark blue) which is located on the domain connection (dark blue). When the amino acid variant at the candidate site 173 is Gln, a hydrogen bond (magenta dotted line, panel (D)) is predicted between Gln173 and Lys297 by the DeepView/Swiss-PdbViewer.

To examine the possible functional importance of the two selection-candidate amino acid sites in PGIC1, we homology-modeled the dimeric protein structure of the translated amino acid sequence of *PgiC1*. The homology-modeled PGIC1 protein structure for *F*. *ovina* in the present study is closely similar to the structure reported in earlier studies of PGI (e.g. [31]), with only 0.45 Å root-mean-square deviations for the backbone atoms from the template Toxoplasma 3ujh.pdb structure. Within the functional, dimeric PGI unit (see Fig 1A), each of the two monomers contains two main regions (the “small” and “large” domains [32], [33], corresponding, respectively, to amino acid sites 114–290 and 317–509 in *F*. *ovina* PGIC1) (Fig 1B). The active site, where the substrate binds and the isomerization reaction takes place, is partially located in the slight cleft between the large and small domains in each monomer [34] (Fig 1B).

On the basis of the locations of the candidate sites in the homology-modeled PGIC1 3-D structure, it can be predicted that amino acid changes at the sites are likely to influence the inter-monomer interaction or the packing of the two domains within each monomer.

Site 200 is located on the edge of the inter-monomer boundary (Fig 1A), and is close to two basic residues (Fig 1C): Lys199 is located on the same monomer as site 200, whereas Lys179 is located on the other monomer. The presence of the acidic residue Asp (as opposed to the non-charged Asn and Gly, Table 3) at candidate site 200 is expected to result in inter-monomer charge-charge interactions with the basic residues Lys199 and Lys179 that may be important for the stability of the PGIC1 dimeric complex. The electrostatic attraction between Asp200 and Lys179 is likely to confer dimeric stability by compensating for the repulsion between Lys179 and Lys199.

The location of site 173 is on the edge of the slight cleft between the two domains within each PGIC1 monomer (Fig 1B): the site is situated within the small domain, close to Lys297 (Fig 1D and 1E) which is found on the interconnecting polypeptide between the two domains of a PGIC1 monomer. The polymorphism at site 173 involves Gln and Glu (Table 3). Whereas a hydrogen bond between Gln173 and Lys297 (Fig 1D) is predicted by the DeepView-Swiss-PdbViewer, no such bond is predicted with Glu173 (Fig 1E), although an electrostatic attractive interaction may occur between Glu173 and Lys297. Both the alternative residues at site 173 interact with Lys297 and differences in the strength of their predicted interactions with Lys297 may have important consequences for domain-domain packing.

**Fig 2:**
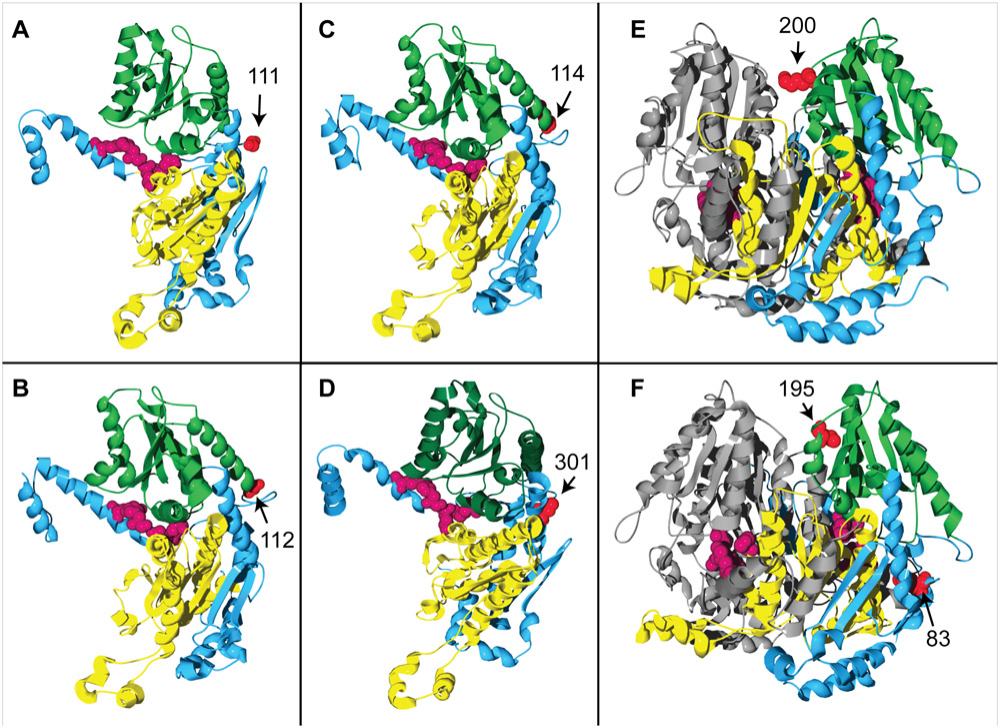
3-D structural locations of PGI amino acid sites in a range of organisms. In panels (A)-(E), the amino acid sites shown in red are those that have been proposed as the potential targets of positive/balancing selection in (A) *Melitaea cinxia* (site 111 [14]), (B) *Dioscorea tokoro* (site 112 [9]), (C) *Arabidopsis thaliana* (site 114 [38]), (D) *Tigriopus californicus* (site 301 [12]) and (E) *Leavenworthia stylosa* (site 200 [13]): the sites are located on the edge of either the inter-monomer boundary or the inter-domain cleft. In panel (F), amino acid sites shown in red are those at which mutation has been shown to significantly alter the activity of PGI in *Homo sapiens* [36] (for the sake of simplicity, only two of the four sites listed in Table 4 are shown here: site 83 is located close to the edge of the inter-domain cleft, while site 195 is located on the edge of the inter-monomer boundary). The four most conserved residues in the active site [35] are indicated in dark magenta in all the panels. The small and large domains, in the PGI monomers in panels (A)-(D) and in one of the two PGI monomers in panels (E) and (F), are shown in dark green and yellow, respectively; the remaining monomer in each of panels E and F is shown in grey.

For comparative purposes, the 3-D protein structural locations of the PGI amino acid sites that have been proposed as candidate targets of positive/balancing selection in previous molecular evolutionary studies of the *Pgi* gene are summarized in Table 4. More than a third (5 out of 12) of the proposed selected sites have locations that are similar to those of the candidate sites in *F*. *ovina* (Table 4, Fig 2). Four human PGI amino acid sites, mutations at which have been shown, by biochemical studies, to significantly change the stability and/or kinetics of the PGI enzyme [36], [37], also have locations that are similar to those of the candidate sites identified in the present study (Table 4, Fig 2).

**Table 4:** 3-D structural locations of PGI amino acid sites in a range of organisms.

Species	Amino acid site	Location in the PGI 3-D protein structure^c^	PGI 3-D protein structure^e^
*Arabidopsis thaliana*	114^a^ [38]	Edge of the cleft between the large and small domains	Fig 2C, modeled in the present study
*Leavenworthia stylosa*	200^a^ [13]	Edge of the inter-monomer boundary	Fig 2E, modeled in the present study^f^
*Dioscorea tokoro*	112^a^ [9]	Edge of the cleft between the large and small domains	Fig 2B, modeled in the present study
	238^a^ [9]	Surface of the small domain	Fig 2B
*Melitaea cinxia*	111^a^ [14]	Edge of the cleft between the large and small domains	Fig 2A, modeled in the present study
	372^a^, 375^a^ [11]	Interpenetrating loop across interface between monomers^d^	^—^ ^d^
*Tigriopus californicus*	66^a^, 77^a^ [12]	N-terminus	Fig 2D, modeled in the present study
	301^a^ [12]	Edge of the cleft between the large and small domains	Fig 2D
*Colias eurytheme*	369^a^, 375^a^ [15]	Interpenetrating loop across interface between monomers^d^	^—^ ^d^
*Homo sapiens*	195^b^ [36]	Edge of the inter-monomer boundary	Fig 2F, downloaded from Protein Data Bank (PDB; http://www.pdb.org) (PDB code 1jlh)
	83^b^, 101^b^ [36]	Close to the edge of the cleft between the large and small domains	Fig 2F
	100^b^ [37]	Close to the edge of the cleft between the large and small domains	Fig 2F

^a^Amino acid sites that have been proposed as being potential targets of selection.

^b^Four functionally important human PGI amino acid sites that share similar locations to the two candidate targets of positive selection in *F*. *ovina* are also included in the table [36], [37].

^c^The location of the amino acid site was identified in the present study, but see ^d^

^d^The locations of the amino acid sites 372 and 375 in *M*. *cinxia* and of the sites 369 and 375 in *C*. *eurytheme* were identified in earlier studies [11], [15].

^e^PGI 3-D protein structures that have been used, in the present study, for identifying the locations of the amino acid sites listed in the second column.

^f^Because the majority of the PGI amino acid sequence is not available for *L*. *stylosa*, the homology-modeled PGI 3-D structure from the related *L*. *crassa* was used as a proxy (see Material and Methods).

### Relationships between the candidate targets of positive selection and enzyme electromorphs

In the 11 studied individuals that were shown by enzyme electrophoresis to be heterozygous for PGIC electromorphs (Table 1), it is not possible to unambiguously assign each *PgiC1* sequence to a single PGIC enzyme electromorph on the basis of the predicted net charge of their translated polypeptides. Firstly, because only 96% of the full-length *PgiC1* cDNA is covered by each sequence and, secondly, because of the complication that electromorph phenotypes include enzyme products that may also be coded for by the second locus (*PgiC2*) that codes for PGIC in *F*. *ovina* [20]. However, when we examine the combination of charged/uncharged amino acid residues at the two PGIC1 candidate sites within each translated amino acid sequence, and the PGIC EMs present in the individual to which each sequence belongs, there is a correspondence between the residue combinations at the candidate sites and the PGIC EMs (Table 3).

The translated PGIC1 amino acid sequences with acidic amino acid residues at both sites 173 and 200 are mostly found in individuals with PGIC enzyme electromorph EM 1 (Table 3). For example, amino acid sequences translated from Hap1, which have the acidic Glu at site 173 and the acidic Asp at site 200, are found in individuals 1 (EM phenotype = 1,2) and 9 (EM phenotype = 1,4): these two individuals only share EM 1 (Table 3). Amino acid sequences translated from other haplotypes, which have an acidic residue at either site 173 or 200, but not at both sites, are more often found in individuals containing EM 2 (Table 3). For example, haplotypes Hap2, Hap6, Hap12, Hap15, Hap18 and Hap19 must code for EM 2, because one or two of these six haplotypes occurred in each of the individuals 2, 10, 12 and 15, which are homozygotes for EM 2.

## Discussion

Earlier studies of *F*. *ovina* suggest that PGIC enzyme variation may be involved in the species’ adaptive response to diverse microhabitats [17], [18]. The present study of *PgiC1* cDNA sequences in *F*. *ovina* used two, complementary, types of approach to test for positive selection on PGIC1. Both approaches identified PGIC1 amino acid sites 200 and 173 as candidate targets of positive selection. The polymorphism at both sites 173 and 200 involves charge changes. On the homology-modeled PGIC1 protein structure, the two candidate sites are located on the edge of either the inter-monomer boundary or the inter-domain cleft. Investigation of local homology-modeled PGIC1 structure showed that the charge changes at the candidate sites are likely to influence the inter-monomer interaction or the domain-domain folding. Furthermore, the two candidate target sites for positive selection are the only sites that appear to be diagnostic for the two most common PGIC enzyme electromorphs in *F*. *ovina*, which have earlier been shown to have significant allele frequency differences in different grassland microhabitats. Our results provide support for the suggestion that PGIC1 amino acid sites 200 and 173 in *F*. *ovina* are under positive selection.

### Locations of the candidate selected sites in the homology-modeled PGIC1 3-D protein structure

The two amino acid sites that are identified as candidate targets of positive selection in the present study are not randomly distributed within the PGIC1 protein structure. 3-D protein structure homology modeling in the present study shows that the locations of the two candidate sites in *F*. *ovina* PGIC1 are similar to those of PGI amino acid sites that have either been shown to significantly affect the enzyme activity of PGI or been proposed to be the potential targets of positive/balancing selection in other organisms (e.g. [13], [38]).

#### The structural location of the candidate site 200

**Fig 3:**
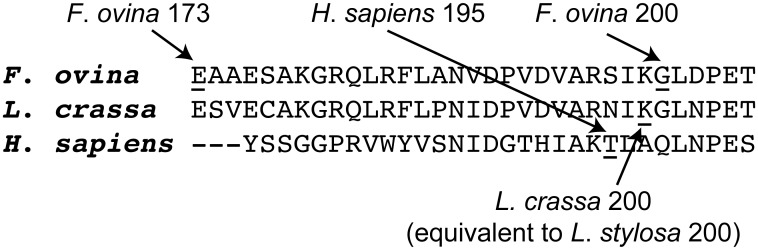
Multi-species PGI amino acid sequence alignment around the *F*. *ovina* candidate targets of positive selection. The multi-species alignment shows that the *F*. *ovina* PGIC1 candidate site 200 is close to (potentially) functionally important sites in other species. The *F*. *ovina* PGIC1 site 200 is next to the *L*. *stysola* PGI site 200 that has been proposed as a candidate target of balancing selection [13], and is just two amino acid residues away from the human PGI site 195, a mutation at which has been shown to significantly reduce the enzyme stability of PGI [36]. The alignment includes PGI amino acid sequences from *F*. *ovina* (Hap 2), *L*. *crassa* (GenBank protein id/gb: AF054455 [39]) and *Homo sapiens* (PDB code/pdb: 1jlh [40]). Because the majority of the PGI amino acid sequence is not available for *L*. *stylosa*, a sequence from the related *L*. *crassa* was used instead.

The candidate site 200 in *F*. *ovina* is located on the edge of the inter-monomer boundary of the PGI dimer. Crystallographic structure analyses show that interactions between the monomers at the inter-monomer boundary are the main forces responsible for the tight association of the two monomers [32], and biochemical analyses show that a mutation at the human PGI amino acid site 195 causes a 39-fold reduction in the thermal stability of PGI [36]. The human PGI amino acid site 195 has a location adjacent to that of the candidate site 200 in *F*. *ovina*, on the inter-monomer boundary (Figs 1A and 2F). The PGI amino acid site 200 in *Leavenworthia stylosa*, which has been proposed as a target of balancing selection [13], also has a closely similar location to that of the candidate site 200 in *F*. *ovina* (Figs 1A and 2E). The similarity between the 3-D structural locations of PGI sites 200 in *F*. *ovina*, 195 in humans and 200 in *L*. *stylosa* is also reflected in the multispecies alignment of PGI amino acid sequences in the present study: PGI sites 195 in humans and 200 in *L*. *stylosa* are two, or less than two, amino acid residues away from the candidate site 200 in *F*. *ovina* (Fig 3).

#### The structural location of the candidate site 173

The candidate site 173 in *F*. *ovina* is located on the edge of the inter-domain cleft within each PGI monomer. The PGI active site is partially located within the inter-domain cleft [34], and mutations at three human PGI amino acid sites (83, 100, 101) that are located near to the *F*. *ovina* candidate site 173 (Figs 1B and 2F, Table 4) have been shown to be functionally important in that they lead to significant changes in the thermal stability and/or kinetic properties of human PGI [36], [37]. PGI amino acid sites 114 in *Arabidopsis thaliana*, 111 in *Melitaea cinxia* and 112 in *Dioscorea tokoro*, which have been proposed as potential targets of selection [9], [14], [38], also have similar locations to that of the candidate site 173 in *F*. *ovina* (Figs 1B,2A, 2B and 2C).

### Charge changes at the candidate sites and relationships between the charge changes and enzyme electromorphs

Earlier studies of enzyme variation in replicated natural populations of *F*. *ovina*, showed that the two most common PGIC electromorphs (EMs 1 and 2) had significantly different frequencies in different grassland microhabitats [17], [18], suggesting that these electromorphs may be involved in local adaptation within the fine-scale habitat mosaic. In the present study, both amino acid sites that are identified as candidate targets of positive selection involve charge-changing polymorphisms, and these two sites are the only sites that appear to be diagnostic for EMs 1 and 2.

Significant correlations between PGI EMs and environmental factors have also been reported in a wide range of other organisms (see the references in [3], [41]). Results from studies of DNA polymorphism in a number of species suggest that there is balancing or positive selection on PGI, and particular amino acid sites have been proposed as possible targets of selection (Table 4). These proposed targets of selection typically involve charge changes and distinguish between PGI EMs, as in the present study.

The potential adaptive significance of the charge-changing amino acid polymorphisms that underlie the variation in PGI EMs has been extensively studied in the butterfly, *Melitaea cinxia* (e.g. [42], [43]). For example, a study by Saastamoinen and Hanski [44] of the single-nucleotide polymorphisms at the codons of the charge-changing amino acid sites (111 and 372) that identify the common PGI electromorph, EM F, in *M*. *cinxia* [14] showed that individuals with genotypes corresponding to EM F had a higher body-surface temperature at low ambient temperatures—allowing them to start flying earlier in the morning than other genotypes. The EM F-genotype females are, therefore, able to start oviposition earlier in the afternoon and produce larger clutch sizes than other genotypes.

In the present study, a combination of evidence from different sources provides support for the suggestion that the PGIC1 amino acid sites 173 and 200, which characterize the PGIC EMs 1 and 2, are under positive selection. Further studies are needed to investigate the potential adaptive significance of the polymorphism at PGIC1 sites 200 and 173 in *F*. *ovina*.

## Material and Methods

### Plant material

Fifteen *F*. *ovina* individuals were collected from five sites covering the full extent of the alvar grasslands on Öland (Table 1). Within sites, soil moisture and pH are the most important determinants of plant community composition [45], and the 15 individuals were chosen to represent the four most extreme combinations of moist, dry/high pH and low pH microhabitats (Table 1). The 15 individuals were also chosen to represent five of the PGIC electromorphs (EMs 1, 2, 4, 5 and 6) that occur most frequently on Öland and which are known to be, at least partly, coded for by *PgiC1* (unpublished data). The study has a particular focus on the two most common electromorphs, EM 1 and EM 2 (Table 1). Neither the study species nor the sampling sites are protected and permission was not required for the collection of the plant material.

### RNA extraction, cDNA synthesis, PCR amplification, cloning and sequencing

Total RNA was extracted from the leaves of each of the 15 *F*. *ovina* individuals using the RNeasy Plant Mini Kit (Qiagen). cDNA was generated from the RNA preparations using the AffinityScript Multiple Temperature cDNA synthesis kit (Agilent Technologies). Ninety six percent of the full-length *PgiC1* cDNA was PCR-amplified using Phusion Hot Start II High-Fidelity DNA Polymerase (Finnzymes) and the primer pair shown in S2 Table. This amplification predominantly detected the *PgiC1* locus but occasionally picked up *PgiC2*. Sequences of *PgiC1* were distinguished from those of *PgiC2* using a phylogenetic analysis, including previously published *PgiC1* and *PgiC2* reference sequences (see S1 Fig for details). The PCR reaction was carried out in a total volume of 50 μl, including 15 μl cDNA and the standard amounts of 5 × Phusion HF Buffer and other reagents. The PCR cycling started with an initial denaturing step at 98°C for 30 s followed by 26 cycles of a denaturing step at 98°C for 10 s, an annealing step at 67°C for 15 s and an extension step at 72°C for 45 s, and ended with a final extension step at 72°C for 10 min. The PCR product was purified with the QIAquick PCR Purification Kit (Qiagen), and ligated into pCR-XL-TOPO vectors and transformed into One Shot TOP10 Chemically Competent *Escherichia coli* cells using the TOPO XL PCR Cloning Kit (Invitrogen). Six to 12 clones from each of the 15 *F*. *ovina* individuals were sequenced in both forward and reverse directions (see S2 Table for primers). The sequencing reactions were performed using the BigDye Terminator v. 1.1 (Applied Biosystems) and analyzed on an ABI 3130*xl* Genetic Analyzer (Applied Biosystems). Nucleotide sequences were assembled and aligned using Sequencer v. 4.7 (Gene Codes Corporation) and MEGA v. 4.0 [46]. The nucleotide diversity (*π*) and Watterson’s estimator of the population mutation rate (*θ*_W_) were calculated using DnaSP v. 5.10.01 [47].

### Tests for positive selection

We used two, complementary, approaches to estimate the *d*N/*d*S ratio (*ω*) (*dN* = nonsynonymous nucleotide substitution rate; *dS* = synonymous substitution rate) for identifying positively selected (*ω* > 1) codon (amino acid) sites within *PgiC1* cDNA sequences. The first (software packages PAML (version 4.5) [29] and HyPhy [27], [28]) is a phylogenetic approach. The second approach (program omegaMap [30]) has a population genetic basis (“population genetic approach”).

The *ω*-ratio test was originally developed for the analysis of highly divergent interspecific sequences [48], [49], where between-sequence differences represent substitutions that have been fixed along independent lineages [50]. Kryazhimskiy and Plotkin [51] and Mugal, Wolf and Kaj [50] show that the *ω*-ratio test may cause bias when analyzing closely related (e.g. conspecific) sequences, where the differences between sequences may represent transient polymorphisms as well as fixed substitutions [50]. In the present study, we attempt to minimize the interference of transient polymorphisms on the *ω*-ratio based selection tests on *F*. *ovina PgiC1*, by combining the phylogenetic and population genetic approaches.

The phylogenetic approach uses only non-identical sequences within a non-recombinant *PgiC1* segment: given the assumption of the PAML *ω*-ratio tests that mutation rate is low [52], the difference between two non-identical, non-recombinant sequences can be regarded as representing fixed substitutions that have accumulated between the sequences. We are not able to judge to what extent the assumption of low mutation rate may be violated in *F*. *ovina PgiC1*. If the mutation rate is high in *F*. *ovina PgiC1*, a high *ω*-ratio for a single codon might reflect a transient polymorphism that is created by the repeated occurrence of new deleterious nonsynonymous mutations that will, with time, be removed by purifying selection.

The population genetic approach complements the phylogenetic approach and has the advantage that it estimates the mutation and recombination rates of the sample and takes these estimates into account when calculating the *ω*-ratio [30]. However, because the population genetic approach uses random sequence samples from a population, a high *ω*-ratio estimated for a single codon using this approach might reflect the over-representation of a single nonsynonymous mutation that occurs as multiple duplicated copies in the sampled sequences.

In the present study, in order to minimize the possible effect of transient polymorphisms on the selection test within *F*. *ovina PgiC1*, we chose to use a conservative strategy. Only sites identified by both the phylogenetic and population genetic approaches were accepted as candidate targets of positive selection.

#### The phylogenetic approach

The phylogenetic approach to the *ω*-ratio test uses codon-based models as implemented in the PAML and HyPhy software packages. The models in HyPhy allow for variation in both the nonsynonymous and synonymous substitution rate among sites, whereas those in PAML only allow for variation in the nonsynonymous substitution rate [53]. All the *ω*-ratio tests in both PAML and HyPhy rely on a prior phylogenetic tree. The phylogenetic trees used in PAML were constructed using PhyML v. 3.0 [54] and in HyPhy using the neighbor-joining algorithm [55] as implemented in the DATAMONKEY web server [27]. A high rate of recombination may interfere with the construction of phylogenetic trees [56] and thus distort the attempts to detect positive selection using phylogeny-based *ω*-ratio tests [57], [58]. To deal with this problem, we first identified the putative recombination breakpoints using the GARD recombination-detection algorithm [59] (available at the DATAMONKEY online server). GARD was run under the best-fitting model of nucleotide substitution, with general discrete substitution rate distribution and two rate classes [60]. We then built the phylogenetic trees and carried out the *ω*-ratio tests on the non-identical sequences [61], [62] within each of the non-recombinant *PgiC1* cDNA sequence segments that were defined on the basis of the recombination breakpoints identified by GARD. In PAML, two nested site models (M1a and M2a; M7 and M8), as implemented in the CODEML program, were used to test for positive selection. In each nested test, the neutral model (M1a/M7) has the restriction *ω* ≤ 1, while the selection model (M2a/M8) adds one more site class with *ω* > 1. A likelihood-ratio test (LRT) was used to test whether the neutral or the selection model better fitted the data in each nested pair (indicating the absence or presence of positive selection on *PgiC1*). Amino acid sites under positive selection were identified using the Bayes Empirical Bayes approach [63]. The REL method in HyPhy was further used to test for positive selection at individual amino acid sites.

#### The population genetic approach

The program omegaMap used in the population genetic approach uses a Bayesian population genetics approximation to the coalescent with recombination [30]. We ran omegaMap twice on the 29 *PgiC1* sequences, each time with 1 000 000 Markov-chain Monte Carlo iterations and thinning every 100 iterations. The first 110 000 iterations were discarded as “burn-ins”. Equal equilibrium frequencies were assumed for all codons, and *ω* and the recombination rate was allowed to vary from codon to codon. A prior run was used to decide the starting values of the model parameters (*ω*, the recombination rate, the transition-transversion ratio, the rate of synonymous transversion, and the rate of insertion/deletion). The remaining model settings follow the recommendations of the software developers. The two runs were checked for convergence before they were merged to infer the posterior distribution of *ω*.

### Protein structure modeling

Homology modeling of the dimeric protein structure of the translated amino acid sequence of *PgiC1* was carried out using the SWISS-MODEL workshop [64]. A PGI crystal structure from *Toxoplasma gondii* (Protein Data Bank (PDB) [65] code 3ujh, 2.10 Å), whose amino acid sequence showed the highest sequence identity (55–56%) to that of *F*. *ovina* PGIC1 sequences, was used as a template structure. The deduced Hap2 amino acid sequence (the most common haplotype in the sampled *F*. *ovina* individuals) was used to model the dimeric PGIC1 protein structure. The ProSA-web server [66] was used to evaluate the overall quality of the modeled PGIC1 dimer by comparing the z-score [67], [68] calculated for the PGIC1 dimer with the z-scores for all the experimental protein structures deposited in PDB. The z-score of -10.16 for the homology-modeled PGIC1 protein structure (which has 544 amino acid residues) falls within the range of z-scores for X-ray determined structures in PDB that have a similar number of residues (S2 Fig), indicating that the quality of the modeled PGIC1 structure is satisfactory. The root-mean-square deviations for the backbone atoms between the homology-modeled *F*. *ovina* PGIC1 protein structure and the template 3ujh.pdb structure was estimated with DeepView/Swiss-PdbViewer v. 4.04 [69], [70].

The polymorphic PGIC1 amino acid sites that were identified as candidate targets of positive selection in the present study were mapped onto the modeled PGIC1 3-D protein structure using DeepView/Swiss-PdbViewer. In order to further investigate the potential functional importance of these polymorphic candidate sites, we used the MUTATE tool in DeepView/Swiss-PdbViewer to predict the local structural changes in the PGIC1 protein that result from the amino acid changes at the candidate sites. For example, the polymorphism at candidate site 173 involves amino acid residues Glu and Gln and, when the MUTATE tool was used to “mutate” the residue Glu173 to Gln173 in the modeled PGIC1 structure, the predicted structural changes after the “mutation” were used to assess the potential functional importance of site 173.

For comparative purposes, the PGI amino acid sites that have been proposed as being under positive/balancing selection in a range of other organisms are summarized in Table 4. The proposed selected sites listed in Table 4 were also mapped onto the PGI 3-D protein structure using DeepView/Swiss-PdbViewer. Human PGI amino acid sites, at which mutations have been shown to significantly affect the activity of the PGI enzyme [36], [37], were also mapped onto the PGI 3-D protein structure in the present study, but only those sites with similar locations to the candidate sites identified in the present study are shown in Table 4.

The same homology-modeling approach that was used to model 3-D protein structures for *F*. *ovina* PGIC1 in the present study was used to identify the locations of the proposed selected amino acid sites in *L*. *stylosa*, *D*. *tokoro*, *A*. *thaliana*, *M*. *cinxia* and *Tigriopus californicus* (see Table 4). No experimental PGI 3-D protein structures are available for these five species and not all the proposed selected amino acid sites listed for these species in Table 4 have had their locations determined by homology-modeling in earlier studies. The GenBank protein id of the amino acid sequences used for modeling the 3-D protein structures in *D*. *tokoro*, *A*. *thaliana*, *M*. *cinxia* and *T*. *californicus* are, respectively, BAA23185 [9], BAB17654 [38], ACF57689 [14] and AFN42997 [12]. Because the majority of the PGI amino acid sequence is not available for *L*. *stylosa*, a sequence (AAC08411 [39]) from the related species, *L*. *crassa*, was used for the homology-modeling. ProSA-web z-scores for the five additional 3-D protein structures modeled in the present study range between -11.22 and -9.63. All five z-scores fall within the ranges of those for X-ray determined protein structures, with equivalent numbers of residues, in PDB (S2 Fig)—indicating that the modeled PGI structures have a satisfactory quality. These five additional 3-D protein structures modeled in the present study have 0.75-Å, or less, root-mean-square deviations for the backbone atoms from the template structures. The PDB codes for the template structures are 3ujh for *L*. *crassa*, *D*. *tokoro* and *A*. *thaliana*, and 1gzd for *M*. *cinxia* and *T*. *californicus*.

## Supporting Information

S1 Fig Maximum likelihood tree of the 36 *PgiC* expressed sequence variants (S1 Table) from *F*. *ovina*. Sequence variants Nos.1-22 (S1 Table) are identified by the codes for the corresponding haplotypes (Hap1—Hap22; S1 Table); the remaining sequence variants are identified by numbers (Nos. 23–36). The ML tree was inferred using PhyML software [54]: indels were not considered. Only bootstrap values larger than 50 are shown. Four earlier published *F*. *ovina PgiC1* sequences and one *F*. *ovina PgiC2* sequence, as well as one *PgiC* sequence from each of *Bromus sterilis*, *Poa palustris* and *F*. *altissima* (GenBank acc. nos., in order, are DQ225734, DQ225732, DQ22735 and DQ225731, HQ616105, DQ225730, HQ616102, DQ225740) were also included in the analysis. *B*. *sterilis* was used as an outgroup. All the 36 sequence variants group together with the four *F*. *ovina PgiC1* sequences (indicated by arrows) into one well-supported cluster with a bootstrap value of 100 (indicated by bold, italic text), while the *F*. *ovina PgiC2* (indicated by a star) forms a separate, well-supported cluster with the *PgiC* sequence from *P*. *palustris*. All the 36 sequence variants thus represent the *PgiC1* locus rather than *PgiC2*.
(TIF)

S2 Fig Plot of ProSA-web z-scores showing the overall quality of the six homology-modeled PGI 3-D protein structures. The black dots shows the z-scores [67], [68] for the PGI protein structures (Figs 1 and 2) that were homology modeled, in the present study, for (A) *F*. *ovina*, (B) *Melitaea cinxia*, (C) *Dioscorea tokoro*, (D) *Arabidopsis thaliana*, (E) *Leavenworthia crassa* and (F) *Tigriopus californicus*. In each panel, the dark blue and light blue dots show, respectively, the z-scores for all protein structures determined by nuclear magnetic resonance spectroscopy and X-ray analysis and deposited in Protein Data Bank (PDB) [65]. The z-scores for the six homology-modeled PGI structures in the present study fall within the ranges of those for X-ray determined protein structures in PDB that have equivalent numbers of residues.
(TIF)

S1 File Identification of PgiC1 cDNA sequence variants (haplotypes). (DOCX)

S1 Table The distribution of the 36 sequence variants identified among 113 sequenced clones, originating from the 15 studied *F*. *ovina* individuals. (DOCX)

S2 Table Primers used for PCR amplification and sequencing of *PgiC1* cDNA derived from *F*. *ovina* individuals. (DOCX)
