# Supplementary figures and images for: Evidence for Positive Selection within the PgiC1 Locus in the Grass Festuca ovina
Source: PLoS One. 2015 May 6;10(5):e0125831. doi: 10.1371/journal.pone.0125831 (PMC4422690; doi:10.1371/journal.pone.0125831)

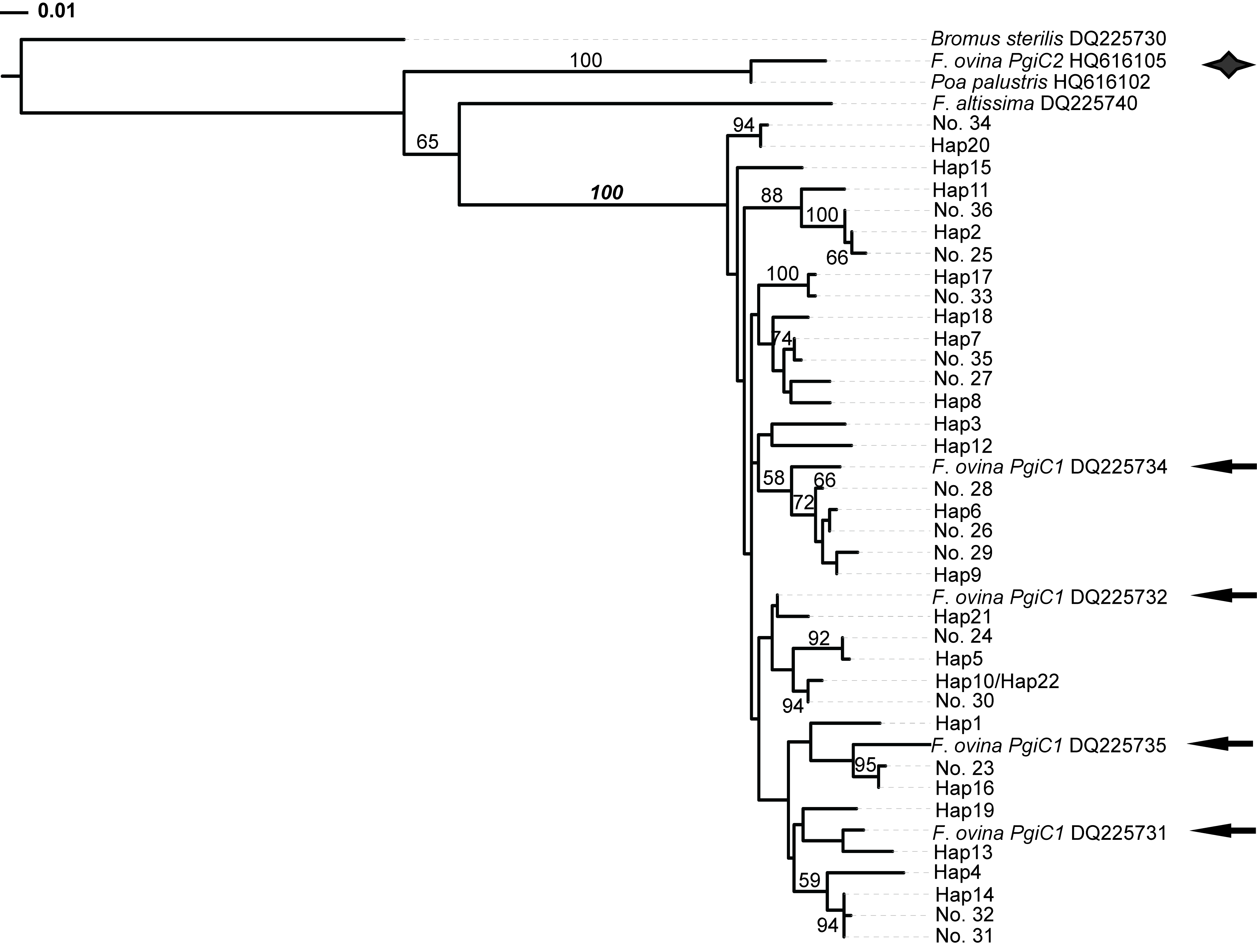

Supplement: S1 Fig — Sequence variants Nos.1-22 (S1 Table) are identified by the codes for the corresponding haplotypes (Hap1—Hap22; S1 Table); the remaining sequence variants are identified by numbers (Nos. 23–36). The ML tree was inferred using PhyML software [54]: indels were not considered. Only bootstrap values larger than 50 are shown. Four earlier published F. ovina PgiC1 sequences and one F. ovina PgiC2 sequence, as well as one PgiC sequence from each of Bromus sterilis, Poa palustris and F. altissima (GenBank acc. nos., in order, are DQ225734, DQ225732, DQ22735 and DQ225731, HQ616105, DQ225730, HQ616102, DQ225740) were also included in the analysis. B. sterilis was used as an outgroup. All the 36 sequence variants group together with the four F. ovina PgiC1 sequences (indicated by arrows) into one well-supported cluster with a bootstrap value of 100 (indicated by bold, italic text), while the F. ovina PgiC2 (indicated by a star) forms a separate, well-supported cluster with the PgiC sequence from P. palustris. All the 36 sequence variants thus represent the PgiC1 locus rather than PgiC2. (TIF) [file pone.0125831.s001.tif]

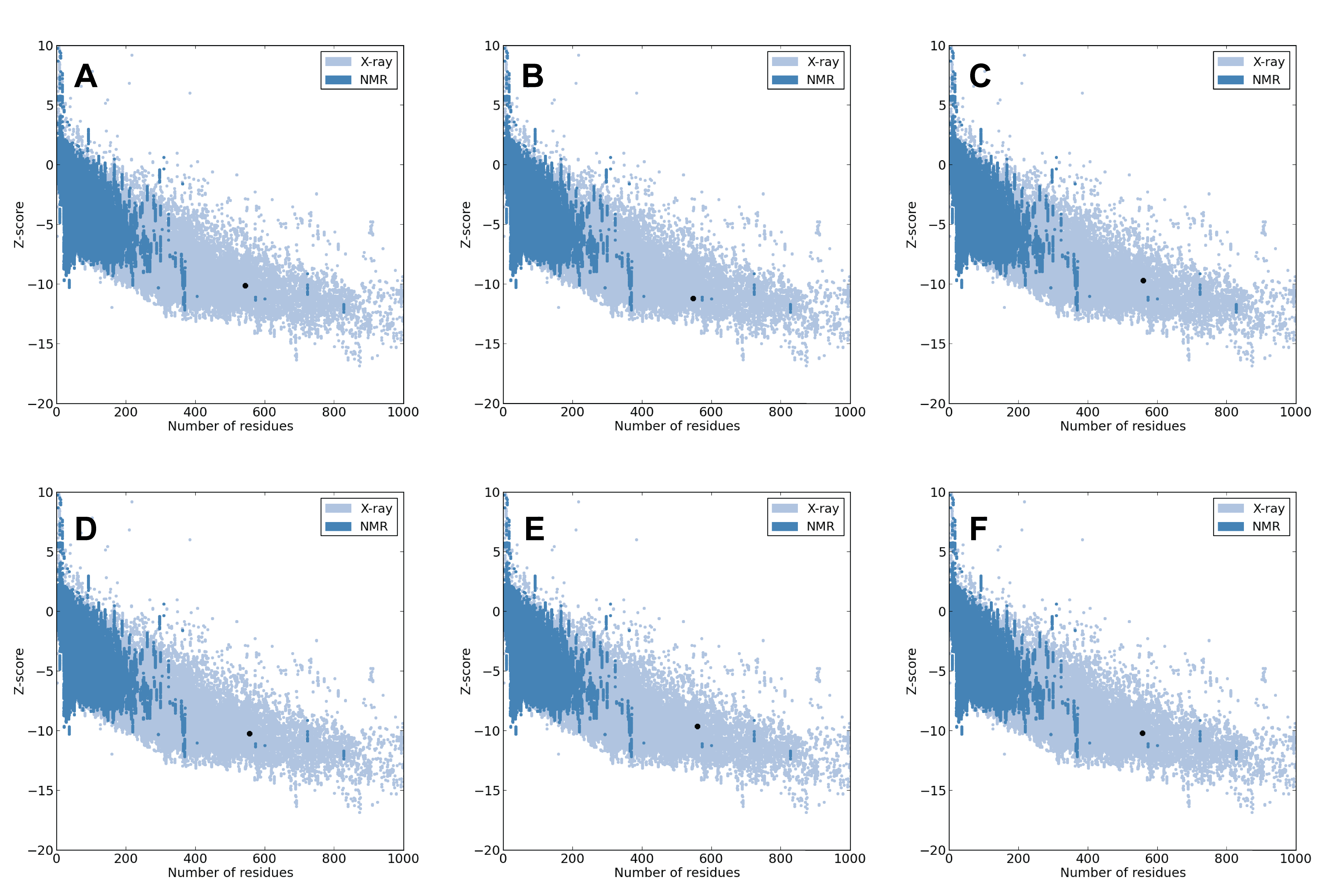

Supplement: S2 Fig — The black dots shows the z-scores [67], [68] for the PGI protein structures (Figs 1 and 2) that were homology modeled, in the present study, for (A) F. ovina, (B) Melitaea cinxia, (C) Dioscorea tokoro, (D) Arabidopsis thaliana, (E) Leavenworthia crassa and (F) Tigriopus californicus. In each panel, the dark blue and light blue dots show, respectively, the z-scores for all protein structures determined by nuclear magnetic resonance spectroscopy and X-ray analysis and deposited in Protein Data Bank (PDB) [65]. The z-scores for the six homology-modeled PGI structures in the present study fall within the ranges of those for X-ray determined protein structures in PDB that have equivalent numbers of residues. (TIF) [file pone.0125831.s002.tif]
