# Supplementary material for: Evidence for Positive Selection within the PgiC1 Locus in the Grass Festuca ovina
Source: PLoS One. 2015 May 6;10(5):e0125831. doi: 10.1371/journal.pone.0125831 (PMC4422690; doi:10.1371/journal.pone.0125831)
Supplement: S1 File — (DOCX) [file pone.0125831.s003.docx]

**S1 File. Identification of *PgiC1* cDNA sequence variants (haplotypes).**

*PgiC1* cDNA from each studied individual was PCR amplified, ligated into the pCR-XL-TOPO cloning vector (Invitrogen), and subsequently transformed into One Shot TOP10 Chemically Competent *Escherichia coli* cells (Invitrogen). A total of 113 clones, originating from the 15 studied individuals, was isolated and sequenced, and 36 sequence variants were identified (Nos. 1-36; S1 Table). Phylogenetic analysis showed that all of these variants grouped together with previously published *F*. *ovina* *PgiC1* sequences into one well-supported cluster (S1 Fig.) – indicating that they all represent *PgiC1*. Because *PgiC1* is a single-copy gene (e.g. [[1](#_ENREF_1)]), and Öland material of *F. ovina* is diploid [[2](#_ENREF_2)], a maximum of two *PgiC1* cDNA sequence variants are expected to be present in each analyzed individual. However, eight out of the 15 analyzed individuals contained more than two *PgiC1* sequence variants (S1 Table), indicating the presence of PCR artifacts arising either as a result of DNA-polymerase introduced errors (e.g. mis-incorporation of individual nucleotides [[3](#_ENREF_3)]) or the formation of chimeric PCR products [[4](#_ENREF_4)]. PCR artifacts should occur at a lower frequency than “true” *PgiC1* cDNA sequences, especially under strict PCR conditions, which is the case in the present study: we used Phusion Hot Start II High-Fidelity DNA Polymerase (Finnzymes) and a limited number (26) of PCR cycles. We therefore regarded 14 sequence variants (Nos. 23-36; S1 Table) that occurred only once among the 113 sequenced clones as PCR artifacts (cf. [[5](#_ENREF_5)]), and excluded them from further analysis. Sequence variants (Nos. 1-18, Nos. 20-22; S1 Table) that were found twice or more were accepted as *PgiC1* haplotypes (Hap1-Hap18, Hap20-Hap22; S1 Table). Sequence variant No. 19, which occurred only once in the material, was also accepted as a *PgiC1* haplotype (Hap19; S1 Table). The possibility that sequence variant No. 19 represents an erroneous chimeric sequence can be excluded, because the formation of chimeras requires at least two different templates, and a minimum of three sequence variants will be present in an individual with a PCR chimera. The possibility that sequence variant No. 19 was derived from variant No. 18 as a result of polymerase-introduced errors within Individual 12 can also be excluded, because the sequence distance between these two variants was 0.011 (equal to 18 nt differences). Such a high level of pairwise sequence difference is unlikely to result solely from DNA polymerase-induced errors: the error rate for the Phusion Hot Start II High-Fidelity DNA Polymerase that was used for the PCR reactions is 4.4 × 10-7 per base per duplication (<https://extranet.fisher.co.uk/webfiles/fr/Pjointes/Documentation/FNZ003_EN%20phusion_high_fidelity_dna_polymerases.pdf>https://extranet.fisher.co.uk/webfiles/fr/Pjointes/Documentation/FNZ003_EN%20phusion_high_fidelity_dna_polymerases.pdf).

The 30 *PgiC1* sequences from the 15 studied individuals belong to 22 *PgiC1* haplotypes (Hap1-Hap22). The two *PgiC1* sequences within each of the 15 diploid *F*. *ovina* individuals (ind.s) belong, respectively, to haplotypes Hap1 & Hap2 (ind. 1), Hap2 & Hap12 (ind. 2), Hap13 & Hap14 (ind. 3), Hap2 & Hap5 (ind. 4), Hap10 & Hap11 (ind. 5), Hap8 & Hap9 (ind. 6), Hap6 & Hap7 (ind. 7), Hap2 & Hap4 (ind. 8), Hap1 & Hap3 (ind. 9), Hap15 & Hap22 (ind. 10), Hap16 & Hap17 (ind. 11), Hap18 & Hap19 (ind. 12), Hap7 & Hap20 (ind. 13), Hap2 & Hap21 (ind. 14), Hap6 & Hap15 (ind. 15) (S1 Table).

**References**

1. Ghatnekar L. A polymorphic duplicated locus for cytosolic PGI segregating in sheep's fescue (*Festuca ovina* L.). Heredity (Edinb). 1999;83: 451-459.

2. Turesson G. Studien über *Festuca ovina* L. II. Chromosomenzahl und viviparie. Hereditas. 1930;13: 177-184.

3. Keohavong P and Thilly WG. Fidelity of DNA polymerases in DNA amplification. Proc Natl Acad Sci USA. 1989;86: 9253-9257.

4. Saiki RK, Gelfand DH, Stoffel S, Scharf SJ, Higuchi R, Horn GT, et al. Primer-directed enzymatic amplification of DNA with a thermostable DNA polymerase. Science. 1988;239: 487-491.

5. Strandh M, Westerdahl H, Pontarp M, Canbäck B, Dubois MP, Miquel C, et al. Major histocompatibility complex class II compatibility, but no class I, predicts mate choice in a bird with highly developed olfaction. Proc R Soc Lond B Biol Sci. 2012;279: 4457-4463.
