# Supplementary material for: Evidence for Positive Selection within the PgiC1 Locus in the Grass Festuca ovina
Source: PLoS One. 2015 May 6;10(5):e0125831. doi: 10.1371/journal.pone.0125831 (PMC4422690; doi:10.1371/journal.pone.0125831)
Supplement: S1 Table — (DOCX) [file pone.0125831.s004.docx]

**S1 Table.** **The distribution of the 36 sequence variants identified among 113 sequenced clones, originating from the 15 studied *F*. *ovina* individuals.**

|  | |  |  | Individual | | | | | | | | | | | | | | | Sum^b^ |
| --- | --- | --- | --- | --- | --- | --- | --- | --- | --- | --- | --- | --- | --- | --- | --- | --- | --- | --- | --- |
| Haplotype | | Sequence variant^a^ |  | 1 | 2 | 3 | 4 | 5 | 6 | 7 | 8 | 9 | 10 | 11 | 12 | 13 | 14 | 15 |  |
| Hap1 | No. 1 | |  | 6 | - | - | - | - | - | - | - | 2 | - | - | - | - | - | - | 8 |
| Hap2 | No. 2 | |  | 2 | 4 | - | 2 | - | - | - | 2 | - | - | - | - | - | 2 | - | 12 |
| Hap3 | No. 3 | |  | - | - | - | - | - | - | - | - | 4 | - | - | - | - | - | - | 4 |
| Hap4 | No. 4 | |  | - | - | - | - | - | - | - | 6 | - | - | - | - | - | - | - | 6 |
| Hap5 | No. 5 | |  | - | - | - | 6 | - | - | - | - | - | - | - | - | - | - | - | 6 |
| Hap6 | No. 6 | |  | - | - | - | - | - | - | 7 | - | - | - | - | - | - | - | 5 | 12 |
| Hap7 | No. 7 | |  | - | - | - | - | - | - | 2 | - | - | - | - | - | 2 | - | - | 4 |
| Hap8 | No. 8 | |  | - | - | - | - | - | 3 | - | - | - | - | - | - | - | - | - | 3 |
| Hap9 | No. 9 | |  | - | - | - | - | - | 4 | - | - | - | - | - | - | - | - | - | 4 |
| Hap10 | No. 10 | |  | - | - | - | - | 4 | - | - | - | - | - | - | - | - | - | - | 4 |
| Hap11 | No. 11 | |  | - | - | - | - | 2 | - | - | - | - | - | - | - | - | - | - | 2 |
| Hap12 | No. 12 | |  | - | 2 | - | - | - | - | - | - | - | - | - | - | - | - | - | 2 |
| Hap13 | No. 13 | |  | - | - | 2 | - | - | - | - | - | - | - | - | - | - | - | - | 2 |
| Hap14 | No. 14 | |  | - | - | 4 | - | - | - | - | - | - | - | - | - | - | - | - | 4 |
| Hap15 | No. 15 | |  | - | - | - | - | - | - | - | - | - | 4 | - | - | - | - | 1 | 5 |
| Hap16 | No. 16 | |  | - | - | - | - | - | - | - | - | - | - | 2 | - | - | - | - | 2 |
| Hap17 | No. 17 | |  | - | - | - | - | - | - | - | - | - | - | 2 | - | - | - | - | 2 |
| Hap18 | No. 18 | |  | - | - | - | - | - | - | - | - | - | - | - | 5 | - | - | - | 5 |
| Hap19 | No. 19 | |  | - | - | - | - | - | - | - | - | - | - | - | 1 | - | - | - | 1 |
| Hap20 | No. 20 | |  | - | - | - | - | - | - | - | - | - | - | - | - | 4 | - | - | 4 |
| Hap21 | No. 21 | |  | - | - | - | - | - | - | - | - | - | - | - | - | - | 5 | - | 5 |
| Hap22 | No. 22 | |  | - | - | - | - | - | - | - | - | - | 2 | - | - | - | - | - | 2 |
| - | No. 23 | |  | - | - | - | - | - | - | - | - | - | - | 1 | - | - | - | - | 1 |
| - | No. 24 | |  | - | - | - | 1 | - | - | - | - | - | - | - | - | - | - | - | 1 |
| - | No. 25 | |  | - | - | - | 1 | - | - | - | - | - | - | - | - | - | - | - | 1 |
| - | No. 26 | |  | - | - | - | - | - | - | 1 | - | - | - | - | - | - | - | - | 1 |
| - | No. 27 | |  | - | - | - | - | - | - | 1 | - | - | - | - | - | - | - | - | 1 |
| - | No. 28 | |  | - | - | - | - | - | - | 1 | - | - | - | - | - | - | - | - | 1 |
| - | No. 29 | |  | - | - | - | - | - | 1 | - | - | - | - | - | - | - | - | - | 1 |
| - | No. 30 | |  | - | - | - | - | 1 | - | - | - | - | - | - | - | - | - | - | 1 |
| - | No. 31 | |  | - | - | 1 | - | - | - | - | - | - | - | - | - | - | - | - | 1 |
| - | No. 32 | |  | - | - | 1 | - | - | - | - | - | - | - | - | - | - | - | - | 1 |
| - | No. 33 | |  | - | - | - | - | - | - | - | - | - | - | 1 | - | - | - | - | 1 |
| - | No. 34 | |  | - | - | - | - | - | - | - | - | - | - | - | - | 1 | - | - | 1 |
| - | No. 35 | |  | - | - | - | - | - | - | - | - | - | - | - | - | 1 | - | - | 1 |
| - | No. 36 | |  | - | - | - | - | - | - | - | - | - | - | - | - | - | 1 | - | 1 |
| Sum^c^ |  | |  | 8 | 6 | 8 | 10 | 7 | 8 | 12 | 8 | 6 | 6 | 6 | 6 | 8 | 8 | 6 | 113 |

**NOTES.—**The table shows the number of clones within which each of the sequence variants was detected in each individual.

^a^The first 22 of the sequence variants (Nos. 1-22) correspond to the 22 identified *PgiC1* haplotypes (Hap1 - Hap22, see S1 File).

^b^The total number of sequenced clones (among 15 *F*. *ovina* individuals) within which each sequence variant was detected.

^c^The total number of clones that was sequenced for each individual.
