# Supplementary material for: Evidence for Positive Selection within the PgiC1 Locus in the Grass Festuca ovina
Source: PLoS One. 2015 May 6;10(5):e0125831. doi: 10.1371/journal.pone.0125831 (PMC4422690; doi:10.1371/journal.pone.0125831)
Supplement: S2 Table — (DOCX) [file pone.0125831.s005.docx]

**S2 Table. Primers used for PCR amplification and sequencing of *PgiC1* cDNA derived from *F. ovina* individuals.**

| **Primers**^a^ | **Primer sequences** | **Locations** |
| --- | --- | --- |
| *mPgiC_f* | 5’-TCATCTCCGACACCGACCAGT-3’ | exon 1 |
| *mPgiC_r* | 5’-AGATACCGTGCCAGCAGACT-3’ | exon 22 |
| *1e8r* | 5’-GATTACCAAGAGTGGTTTCAGGATC-3’ | exon 8 |
| *622r* | 5’-CAAGAGTGGTTTCAGGAT-3’ | exon 8 |
| *PgiC2_3_e6f* | 5’-CGGTTGGAATTGGTGGTAGCTTC-3’ | exon 6 |
| *PgiC1_4_e16r* | 5’-TCACCAGCCTCATAAGGAAGTC-3’ | exon 16 |
| *me16f* | 5’-AGAGTAACGGAAAGGGTGTC-3’ | exon 16 |
| *M13_Reverse*^b^ | 5’-CAGGAAACAGCTATGAC-3’ | vector^c^ |
| *M13_Forward*^b^ | 5’-GTAAAACGACGGCCAG-3’ | vector^c^ |
| *1e7r* | 5’-TTTGGCGGATTCTGCTGCTT-3’ | exon 7 |

**NOTES.—***mPgiC_f* and *mPgiC_r* comprise the primer pair used to amplify the cDNA of *PgiC1*. All ten primers were used for sequencing.

^a^“f” and “r” within the primer names indicate forward and reverse primers, respectively.

^b^The sequences of the *M13*-*Reverse* and *M13*-*Forward* were provided by Invitrogen (TOPO® XL PCR Cloning Kit).

^c^pCR-XL-TOPO vector (Invitrogen) used for cloning.
